# Performance Analysis of Receive Diversity in Wireless Sensor Networks over GBSBE Models

**DOI:** 10.3390/s101211021

**Published:** 2010-12-03

**Authors:** Shivali Goel, Jemal H. Abawajy, Tai-hoon Kim

**Affiliations:** 1 School of Engineering and Information Technology, Deakin University, Pigdons Road, Geelong, Victoria, 3217, Australia; E-Mail: sgoe@deakin.edu.au; 2 School of Engineering and Information Technology, Deakin University, Pigdons Road, Geelong, Victoria, 3217, Australia; E-Mail: jemal@deakin.edu.au; 3 Department of Multimedia Engineering, Hannam University, 133 Ojeong-dong, Daedeok-gu, Daejeon, 306-791, Korea

**Keywords:** geometrically based single bounce elliptical model, wireless sensor networks, smart antennas, receive diversity

## Abstract

Wireless sensor networks have attracted a lot of attention recently. In this paper, we develop a channel model based on the elliptical model for multipath components involving randomly placed scatterers in the scattering region with sensors deployed on a field. We verify that in a sensor network, the use of receive diversity techniques improves the performance of the system. Extensive performance analysis of the system is carried out for both single and multiple antennas with the applied receive diversity techniques. Performance analyses based on variations in receiver height, maximum multipath delay and transmit power have been performed considering different numbers of antenna elements present in the receiver array, Our results show that increasing the number of antenna elements for a wireless sensor network does indeed improve the BER rates that can be obtained.

## 1. Introduction

Advances in directional antennas provide potential benefits in solving various problems in wireless sensor networks (WSNs). A WSN is a network of wirelessly interconnected devices, called sensor nodes, which are able to ubiquitously collect/retrieve data to be sent to a far receiver. Hundreds of nodes are scattered randomly throughout over a wide area, which assemble together, establish a routing topology, and transmit data back to a common collection point [1]. The main features of such networks are high density of nodes, low-mobility, severe power constraints, and high correlation of data among the nodes and also that the nodes can act both as a sensor and as a router towards a centralized node through multi-hop technique. With the development of new wireless technologies and a growing demand for miniaturized, low-powered, low-cost yet simpler and reasonably efficient wireless communication devices, there has been a growing interest in WSNs for a wide variety of applications ranging from seismic studies and life sciences, security-sensitive applications, social, military, and environmental problems.

Wireless communication involves entire environment related effects on the propagated signals between the transmitter and the receiver. Conventional communication systems suffer from multipath signals, Doppler spread and high propagation delays. Due to the irregular distribution of scatterers present in the environment, multipath signals arrive at the receiver from different directions at different times. All of these multipaths taken by the wireless signal possess different properties, and hence, each multipath signal has its own distinctive carrier phase shift, amplitude, angle of arrival, and time delay. A possible approach to address these issues is through the geometrical definition of the scattering region to calculate the above parameters. The geometry of the multipath propagation plays a vital role for communication systems to suppress multipath [2]. A GBSBEM for single bounce multipath components involving randomly placed scatterers is presented here.

In this paper, we combine the Geometrically Based Single Bounce Elliptical Model (GBSBEM) with other aspects of fading channel and establish a vector channel model requirement for smart antennas employed at the receiver. Since we are using a cluster-based WSN deployment model, the sensor nodes do not face the reachback problem as they have to transmit the information over a shorter distance to the cluster head, hence they can be designed to work with comparatively lower power. There are benefits to incorporating receive diversity into wireless sensor networks [3]. In addition to correct reception of data at the receiver and hence performance improvement, exploiting diversity techniques at the receiver can help in saving energy substantially and lead to reduced battery consumption, consequently increasing network lifetime.

The rest of the paper is organized as follows. In Section 2, we discuss some of the related work. Section 3 describes the system model and the GBSBE channel model for the proposed system. In Section 4, we discuss the receiver structure exploiting receive diversity followed by various diversity combining techniques at the receiver. In Section 5 we present the simulation results and analyze the performance based on different variables for different number of receive antenna elements. Finally we present our conclusions in Section 6.

## 2. Related Work

Wireless sensor networks have attracted a lot of attention recently. In [4], the average bit-error rate performance of wireless sensor networks based on the generalized approach to signal processing in the presence of noise under the use of multiple antennas at the sensor sink is investigated as a function of the transmit antenna update rate at the sensor nodes when using binary phase-shift keying signals in flat Rayleigh fading channels. In [3], the benefits of incorporating receive diversity into wireless sensor network (WSN) applications that require high data fidelity and resolution upon event triggering is demonstrated. In [5], a cooperative diversity scheme that increases the network lifetime and the communication reliability has been proposed where the identical sensors are randomly scattered over a wide area. These nodes collect a common message and transmit it towards a fusion centre placed in an unarmed air vehicle (UAV). Practically these scenarios face the reachback problem where the nodes are designed with low power transmitters and are often not capable enough to directly transmit data to the far receiver [6].

A distributed algorithm capable of computing linear signal expansions for a sensor broadcast protocol is presented in [7], where each sensor collects the correlated samples, broadcasts a rate-constrained encoding of its samples to every other sensor and forms an estimate of the entire field. To decorrelate, the sensors only need access to samples from a few nearby sensors. Typical applications include collection of data from a remote area. A distributed diversity approach capable of exploiting spatial distribution of sensor nodes has been proposed and analyzed in [8]. However, idealistic assumptions like synchronization and cooperation are introduced to ensure improvement in the network performance.

Other than correct reception of data at the far end receiver and hence performance improvement, exploiting diversity techniques at the receiver can help in saving the energy substantially and leading to reduced battery consumption and subsequently increasing network lifetime. New relaying strategies based on Luby Transform Codes were presented in [9] by exploiting diversity in WSNs. It is understood that the diversity was applied at the transmitter side involving decoding complexity, though light-weight complexity, at the receiver. While the model proposed in [9] required some extra power for performing the encoding and decoding tasks, a cluster based cooperative scheme for multihop WSN was presented in [10] that could minimize the energy consumption of the sensor nodes.

In [11], energy efficiency of a cooperative multiple input single output (MISO) system using two different cluster based models was investigated for a multi-hop WSN. Space-Time Block Coding (STBC) has been used to encode the data, which means more power requirement by the cooperative nodes for the encoding task. The performance of cluster based WSN over GBSBE model has been presented in [12], based on the transmitting power and the varying number of receive antennas. In this paper, we extend the performance based on other quantities like the maximum multipath delay and the receiver height. We have tried to keep the complexity low at the sensor nodes to minimize the amount of power consumed by these nodes. Also, while on one hand we have tried to keep track of the channel properties by using GBSBE Model, on the other hand we have been successful in minimizing the effect of multipaths and fading by exploiting diversity at the receiver.

## 3. System and Channel Model Development

### 3.1. System Model

**Figure 1.**
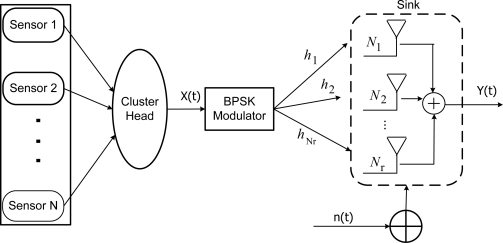
High-Level System Model.

The system model used in this paper for a cluster based WSN architecture for *N_r_* receive antennas is shown in Figure 1. We consider a cluster based WSN architecture with N number of identical sensors deployed over a wide area. The goal is to collect the observations gathered by all the sensors to the cluster head to be transmitted to the receiver. We assume that all the sensors collect the same data and are capable of developing an *ad-hoc* network to disseminate the information among them via efficient flooding. The sensors pass on the information to the cluster head, where this information is filtered and modulated using BPSK and sent to the receiver. Another assumption is that the whole architecture is synchronous and the communication channel between the cluster head and the receiver is subjected to fading, multipath, and noise.

**Figure 2.**
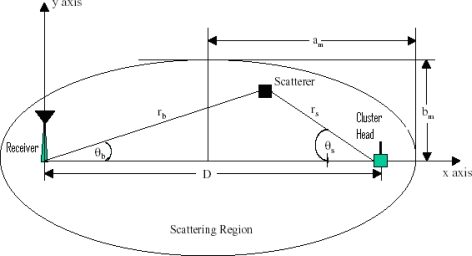
Geometry of the GBSBEM.

When the signal is transmitted, reflections from large objects, diffraction of the waves around objects, and signal scattering dominate the received signal resulting in the presence of multipath components, or multipath signals, at the receiver. Figure 2 depicts a general example of this multipath environment. Each signal component propagates through a different path, determining the amplitude *α_l_*, time delay *τ_l_*, angle of arrival *θ_l_*, the power for the multipath components, and Doppler shift *f_d_* of the *l^th^* multipath signal component. Accordingly, each of these signal parameters will be time-varying [13].

In the GBSBEM, scatterers are uniformly distributed within an ellipse, as shown in Figure 2. An essential attribute of this model is the physical interpretation that only the multipath signals which arrive with an absolute delay ≤ *τ_max_* are accounted. The sensors are placed in such a way that they are surrounded by scatterers and each signal transmitted by each sensor experiences a different multipath environment that determines the amplitude, the time delay, Direction-of-Arrival (DOA), and the power for each multipath component for each sensor.

Considering the distance between the sensor nodes and the receiver to be D, all the scatterers giving rise to single bounce components arriving between time *τ* and *τ* + Δ*τ* lie in the region bounded by the ellipse with semi-major axis, *a_m_* and its semi-minor axis, *b_m_* and are related to the maximum specified delay *τ_max_* as:

(1) $$a_{m}=\frac{{c\tau }_{\text{max}}}{2}$$

(2) $$b_{m}=\frac{1}{2}\sqrt{c^{2}\tau_{\text{max}}^{2}-D^{2}}$$

where c is the speed of propagation. The choice of these parameters is determined by the maximum delay, *τ_max_* of the multipath. Larger values of *τ_max_* imply greater path loss for the multipath and, consequently, lower relative power compared to those with shorter delays.

### 3.2. Channel Model

Let *α_l_* be the complex amplitude of the *l^th^* multipath component and *τ_l_* be the path delay for that component. The complex envelope model for the multipath channel impulse response is given by:

(3) $$h(t)=\sum_{l=1}^{L}\alpha_{l}\delta (t-\tau_{l})$$

where L is the number of the multipath components and is assumed to be the same for all the sensors. Our objective is to determine the values of the amplitude *α_l_*, path delay *τ_l_*, DOA *θ_l_*, the power for the multipath components. We start by determining the distribution of the DOA for a particular multipath component as a function of time-of-arrival.

To simplify the notation, it is convenient to introduce the normalized multipath delay, $\tau_{l}=\frac{c\tau_{l}}{d_{0}}=\frac{\tau_{l}}{\tau_{0}}$, where the distribution of *τ_l_* is given by:

(4) $$f_{r}(r)=\frac{2r^{2}-1}{\beta \sqrt{r^{2}-1}},\;1\;\leq \;r\;\leq \;r_{m}$$

where $\beta =r_{m}\sqrt{r_{m}^{2}-1}$ and $r_{m}=\frac{\tau_{m}}{\tau_{0}}$ is the maximum value of the normalized path delay. Several techniques for selecting *r_m_* are outlined in [2]. A detailed analysis on the pdf of multipath delays, AOA and power spectrum of the elliptical channel model can be found in [14].

The idea is first to define an ellipse corresponding to the maximum multipath delay, *τ_m_* and uniformly placed scatterers inside the ellipse. The relevant signal parameters can then be calculated from the coordinates of the scatterers. It is assumed that the number of multipaths, L and the separation distance between the cluster head and the receiver, D is known. A value of the maximum multipath propagation delay, *τ_m_* is chosen and samples of two uniformly distributed random variables, *x_l_* and *y_l_*, *l*=1,2,…*L* are generated over the interval [−1,1]. These L samples of a random variable are described by the polar coordinates (*r_l_*,*ϕ_l_*)according to the following relationships $r_{l}=\sqrt{x_{l}^{2}+y_{l}^{2}}$ and $\phi_{l}={\text{tan}}^{-1}\left(\frac{y_{l}}{x_{l}}\right)$. These samples are translated so that they are uniformly distributed in an ellipse; the following two transformations are performed:

(5) $$x_{l}=a_{m}r_{l}\text{cos}(\theta_{l})+\frac{D}{2},\;y_{l}=b_{m}r_{l}\text{sin}(\theta_{l})$$

Thus, the multipath propagation distance, *d_l_*, and, the propagation delays, *τ_l_*, can be calculated as $d_{l}=\sqrt{x_{l}^{2}+y_{l}^{2}+\sqrt{{\left(D-x_{l}\right)}^{2}+y_{l}^{2}}}$, and $\tau_{l}=\frac{d_{l}}{c}$, respectively. Following that the receiver system is located at the origin of the coordinate system, the angle of arrivals (AOA) of the multipaths at the receiver are given by $\theta_{l}={\text{tan}}^{-1}\left(\frac{y_{l}}{x_{l}}\right)$.

The power of the direct path component (LOS) can be calculated as below:

(6) $$P_{0}(\text{dBm})=P_{\text{ref}}(\text{dBm})-10n\text{log}\left(\frac{D/c}{d_{\text{ref}}}\right)+G_{t}(\theta_{d})+G_{r}(\theta_{a})$$

where *P_ref_* is the reference power measured at a distance *d_ref_* from the transmitter using omni-directional antennas at the transmitter and the receiver. *P_ref_* can be calculated using Friis’ free space propagation model given by:

(7) $$P_{\text{ref}}(\text{dBm})=P_{T}(\text{dBm})-20\text{log}\left(\frac{4\pi d_{\text{ref}}}{\lambda }\right)$$

where *P_T_* is the transmitted power and *λ*=*c*/*f* is the wavelength for a particular carrier frequency, *f*. The path loss exponent, n typically ranges from 3 to 4 in a microcell environment. *G_t_* (*θ_d_*) *and G_r_* (*θ_a_*) are the gains of the transmit and the receive antennas as functions of the angle of departure, *θ_d_* and the angle of arrival, *θ_a_* respectively. For the LOS component, *θ_d_* and *θ_a_*are both zero. The power of each of the multipath component can be calculated as:

(8) $$P_{l}(dB)=P_{0}(dB)-10n\text{log}(d_{l})-L_{r}+G_{t}(\theta_{d,l})-G_{t}(0)+G_{r}(\theta_{a,l})-G_{r}(0)$$

where *L_r_* is the path loss in dB. Assuming the phase of the multipath components, *γ_l_*, are uniformly distributed over the interval (0,2*π*) the complex amplitudes of the multipath components are calculated as *α_l_* = 10^(*P_l_*−*P*_0_)/20^ *e^jγ_l_^*.

## 4. Receive Diversity

It can be generally supposed that the signal transmitted by the cluster head travels through several resolvable discrete multipaths and arrives at the receiver arrays, each multipath having its own independent DOA, time delay, and amplitude. For example, the sensors are deployed in an open field where they collect data and send to the cluster head. The collected data is sampled and modulated using BPSK modulation and converted into a serial bit stream. This data bit stream needs to be transmitted to the sink to be analyzed.

Assuming that perfect channel state information (CSI) is available at the receiver, if at any time *t*, *s*(*t*) is the transmitted signal across all links, then the transmitted signals are received over *N_r_* independent and identically distributed GBSB channels corrupted by complex Gaussian noise, the received signal *y*(*t*) can be represented as:

(9) $$y(t)=\sum_{l}\alpha_{l}(t)s(t-\tau_{l}(t))+n(t)$$

where *s*(*t*) is the input signal, *n*(*t*) is the additive white Gaussian noise, *α_l_*(*t*) is the attenuation factor for the signal received on the *l^th^* path. As per antenna array theory, each multipath signal brings multiple signals at the receiving array. The effect of every individual multipath signal on every element of the antenna array can be equalized to multiply by *a_k_* (*θ_l_*), known as the steering vector of antenna array where *k* represents the index of antenna array.

For an N-element linear antenna array the channel impulse response of the *i^th^* user can be expressed as:

(10) $$h_{i}(t)=\sum_{l=1}^{L}\alpha_{l,i}(t)a(\theta_{l,i})\delta (t-\tau_{l,i})$$

Thus, the output received at the sink is given by *y*(*t*) = Σ*_l_* *h_i_* (*t*) *s*(*t* − *τ_l_*(*t*)) + *n*(*t*), and the N×1 array response vector or the steering vector *a*(*θ*_*l*,*i*_) is defined as:

(11) $$a(\theta_{l,i})={\left[1e^{-j2\pi \frac{d}{\lambda }\text{sin}(\theta_{l,i}+\Delta_{i})}\ldots e^{-j2\pi \frac{d}{\lambda }(N-1)\text{sin}(\theta_{l,i}+\Delta_{i})}\right]}^{T}$$

where *d* is the element spacing and Δ*_i_* is the angle spread of the *i^th^* user.

The noise on each diversity branch is assumed to be uncorrelated. The collection of independently fading signal branches can be combined in a variety of ways to improve the received SNR. Since the chance of having two deep fades from two uncorrelated signals at any instant is rare, combining them can reduce the effect of the fades. Diversity is a powerful communication receiver technique that provides wireless link improvement at relatively low cost. It exploits the random nature of radio propagation by finding independent signal paths for communication. In virtually all applications, diversity decisions are made by the receiver, and are unknown to the transmitter. The diversity concept can be explained simply. If one radio path undergoes a deep fade, another independent path may have a strong signal. By having more than one path to select from, both the instantaneous and average SNRs at the receiver may be improved. There are a variety of ways in which the independently fading signal branches can be combined; hence, the three most prevalent space diversity-combining techniques used in this paper are the Maximal Ratio Combining (MRC) [15] Equal Gain Combining (EGC) [16,17], and Selection Combining (SC) [18,19].

For example, the received signals are combined at the receiver using MRC to maximize the SNR and give the following expression:

(12) $$\tilde{y}(t)=\sum_{j=1}^{n_{R}}h_{j}^{*}y_{j}(t)=s(t)\sum_{j}^{n_{R}}|h_{j}{|}^{2}+n^{\prime }(t)$$

In terms of the weight vector *w*, where *w*=*h^H^*, the output x at the receiver is given by:

(13) $$\text{x}=\sqrt{E_{s}}h^{H}hs+h^{H}n$$

where $h^{H}h=\sum_{j}^{n_{R}}|h_{j}{|}^{2}$ is the sum of the channel powers across all the receive antennas.

In the presence of channel *h_j_*, the instantaneous SNR, γ_j_, at *j^th^* receive antenna is given by:

(14) $$\gamma_{\text{j}}=\frac{{\left|{\text{h}}_{\text{j}}\right|}^{2}{\text{E}}_{\text{b}}}{{\text{N}}_{0}}$$

where $\frac{E_{b}}{N_{0}}$ is the ratio of the bit energy to noise power spectral density. But since we are equalizing the channel with *h^H^*, with *N* receive antennas, the effective SNR is given by:

(15) $$\gamma_{b_{j}}=\sum_{j=1}^{N_{r}}{\left|h_{j}\right|}^{2}\frac{E_{b}}{N_{0}}$$

(16) $$\gamma_{b_{j}}=N_{r}\gamma_{\text{j}}$$

The received symbols are then passed through a maximum-likelihood detector to produce the estimate of transmitted signal *s̃* (*t*).

## 5. Performance Analysis

In this section we present simulation results to evaluate the performance of our system. We discuss the reliability and robustness of a cluster based WSN system by using smart antennas at the receiver.

### 5.1. Experiment Setup

We used MATLAB to simulate the system. The proposed model has been simulated for a microcell environment. The focus of the model is to consider the scenario of local scattering giving rise to multipaths. These multipaths and the resulting fading are modeled as stochastic processes and channel characteristics like time-variation, amplitude, and angular spread are modeled using GBSBEM.

We consider a cluster-based model with N sensor nodes randomly scattered over a large area. These nodes collect a common message and transmit it towards the cluster head. The information received at the cluster head is filtered and modulated and transmitted to the receiving station. The cluster head is located within a range of 2 meters from this receiving station. In this case both the cluster head and the receiver are surrounded by scatterers and the receiving antenna array is not well above the surrounding objects. The model parameters were chosen to fit the scenario.

Table 1 shows the set of parameters used for simulations to develop the channel and the system model. The transmitted sequence is a BPSK modulated signal and the sequence length is 10^7^. The whole sequence is divided into frames of length 100 symbols and the total number of frames are 10^5^. The channel considered here is a quasi-static channel; *i.e.*, the channel remains constant over the entire frame and changes from one frame to other.

### 5.2. Simulation Result Discussion

We have carried out the simulations where we have different combining schemes at the receiver. We have compared the performance of these schemes with different number of antennas at the receiver. We further analyze the performance of the system by varying the system parameters like receiver height, maximum multipath delay, and transmit power and compare the performance with: (i) no diversity, and (ii) MRC at the receiver.

#### 5.2.1. Performance of the system with different diversity schemes

**Figure 3.**
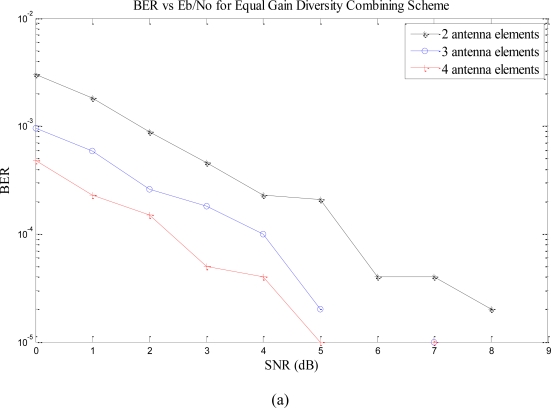

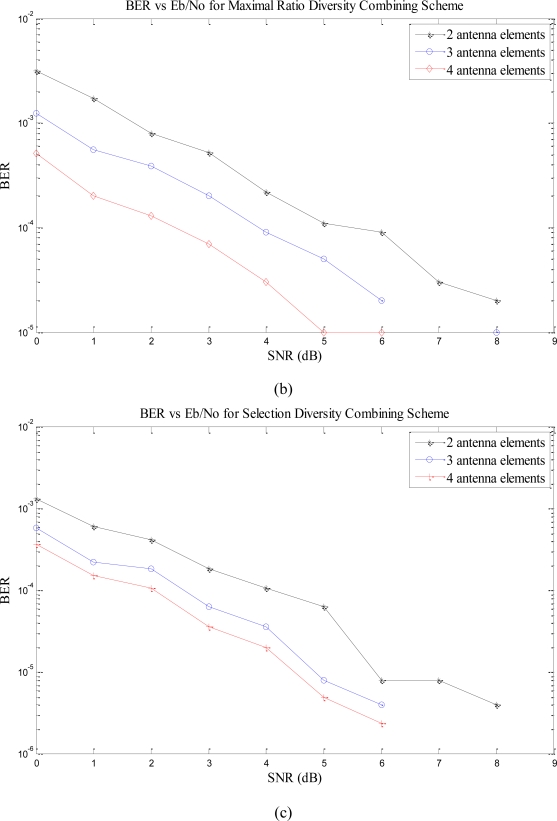
**(a)** BER *vs.* SNR with EGC. **(b)** BER *vs.* SNR with MRC **(c)** BER *vs.* SNR with SC.

We present the performance analyses when we have multiple antennas at the receiver. We apply EGC, SC, and MRC at the receiver to exploit diversity. Figure 3 shows the performance of the system with receive diversity techniques employed at the receiver with 2, 3, and 4 antenna elements. The transmission power is 10 W and all other parameters kept same as in Table 1. Figures 3(a–c) show the performance of EGC, SC, and MRC with different number of antenna elements at the receiver, respectively. The three graphs shows that the performance of the system increase as the number of antenna elements increases.

Table 2 compares the performance of the three receive diversity techniques. The table demonstrates that the BER of the system increases by increasing the antenna elements and decreases with the increase in SNR. At higher SNR, the BER goes to zero.

The performance of EGC is only marginally inferior to MRC. The implementation complexity for EGC is significantly less than the MRC because of the requirement of correct weighing factors. Hence, the basic idea of diversity reception is that, if two or more independent samples of a signal are taken, then these samples will fade in an uncorrelated manner. This means that the probability of all the samples being simultaneously below a given level is much less than the probability of any individual sample being below that level. Thus, a signal composed of a suitable combination of various samples will have much less severe fading properties than any individual sample alone.

#### 5.2.2. Performance of the system with single receives antenna and varying receiver height, maximum multipath delay, and transmits power

In this section we analyze the performance when there is a single antenna in the receive array. The simulations were carried with different varying parameters. First, we perform the simulations based on varying receiver height. If the receiver height is low, there is a possibility that it may suffer from deep fades due to dense environment surrounding the receiver hence degrading the performance of the system. On the other hand, if the receiver is mounted on a higher ground, it will be less susceptible to fading and hence will collect the signal more efficiently.

**Figure 4.**
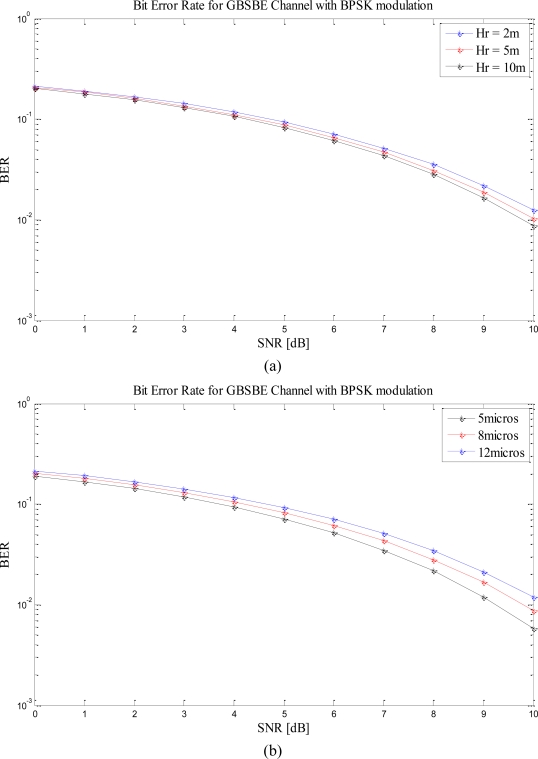

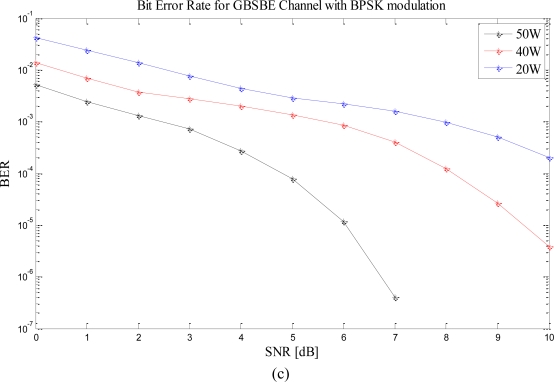
**(a)** BER *vs.* SNR with varying receiver height, *H_R_*(*m*). **(b)** BER *vs.* SNR with varying maximum multipath delay, *τ_max_*(μs). **(c)** BER *vs.* SNR with varying transmit power, *P_T_*(*W*).

Figure 4(a) shows the variations in the BER as the height of the receiver is increased from 2 meters to 10 meters. It can be seen that the receivers closer to ground have higher BER whereas as the height of the receiver is raised from the ground, the BER improves.

Figure 4(b) shows the BER when the maximum multipath delay is varied. As can be seen from Equation (1) and Equation (2) that the semi-major and the semi-minor axis of the ellipse, respectively, is dependent on the maximum multipath delay, τ_max_, therefore, as we change τ_max_, the geometry of the ellipse also changes. The results show that as τ_max_ is decreased, the system gives better performance. It can be explained in terms of the geometry of the ellipse. As τ_max_ increases, a_m_and b_m_ also increases, thus making the ellipse larger, and vice-versa. Larger ellipse means increase in the propagation delays, hence poorer performance. Smaller ellipse means lesser propagation delays, hence better performance.

Figure 4(c) shows the BER performance of the model as a function of SNR under different values of transmission power based on numerical simulation. It can be seen that the use of smart antennas can help significantly in reducing the sensor nodes’ power consumption. However, the performance of the system increases with increase in transmission power. It is evident that the performance of the system varies with variation in transmission power.

#### 5.2.3. Performance of the system with MRC and varying receiver height, maximum multipath delay, and transmit power

**Figure 5.**
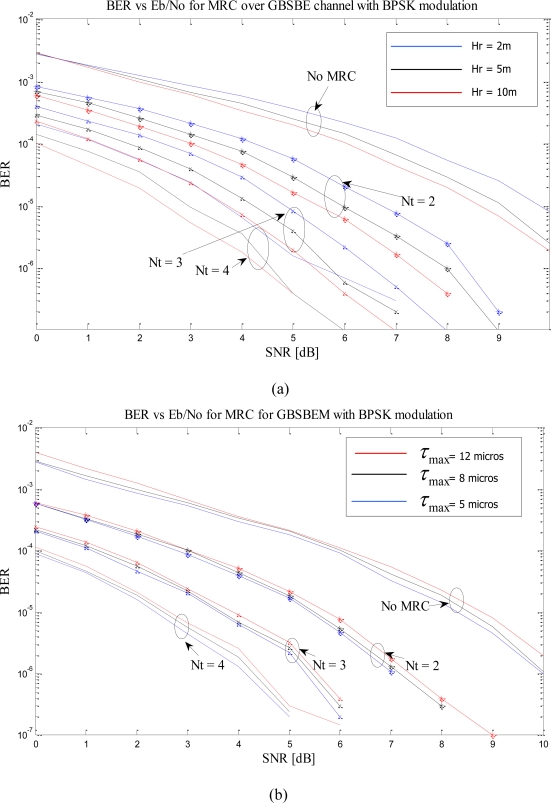
**(a)** BER *vs.* SNR with different receiver height, *H_R_*(*m*) and N_R_ = 1,2,3, and 4. **(b)** BER *vs.* SNR with varying maximum multipath delay, *τ_max_*(μs) and *N_R_*=1,2,3,4.

In this section, we repeat the simulations for different H_R_ and τ_max_ when we have multiple antennas at the receiver. As seen from the table, MRC gives the best performance, thus, for our further simulations we have focused on the system model with MRC at the receiver only and the implementation of EGC and SC is straight forward. Figure 5(a) shows that the performance of the system increase with receiver height irrespective of the number of the receive antenna elements. Figure 5(b) shows the performance based on τ_max_ and proves that BER improves with smaller τ_max_. The simulations have been performed with number of receive antennas up to four but it is not limited and can be extended for higher numbers of receive antennas.

The BER values for multiple receive antennas at different receiver height with different maximum multipath delay have been summarized in Table 3.

## 6. Conclusions

We analyze the problem from the overall performance of the system. The model presented in this paper has been developed for a microcell environment which has a quasi-static channel. A cluster based WSN architecture has been assumed at the transmission side. The cluster head is assumed to be surrounded by local scatterers giving rise to multipath and fading. At the receiver, receiving arrays are used to collect all the multipath components of the signal effectively. The advantage of using smart antennas in a cluster based WSN model has been demonstrated where performance improvements can be realized in terms of received SNR. The numerical simulations based on the variations in receiver height reveal that the performance of the system increases if the receiver height is increased above ground level. Also the numerical simulations based on maximum multipath delay shows that the semi-major and semi-minor axis of the ellipse changes with variations in the maximum multipath delay, hence affecting the performance of the overall system. The performance of the system is also improved as the transmission power increases. Since the cluster head is located very near to the sensor nodes, the sensor nodes do not require high transmission powers so they do not face the reachback problem. The paper justifies the use of receive diversity at the receiver for reliable communication between the cluster head and the receiving arrays and proves that MRC provides the best performance when applying receive diversity. We also quantify the fact that with the increase in the number of antenna elements, we are able to increase the reliability and robustness of the system. The number of antenna elements has been kept low while solving our problem. However, they can be extended to higher number of receive antennas for a large receiving array.

## Figures and Tables

**Table 1.** Parametric Values for the System Model.

**Fixed Parameters**	**Values**
No of frames	100,000
Frame Length	100
Path Loss (Lr)	6 dB
Path loss exponent (n)	3
Number of multipaths (L)	5
Carrier frequency (fc)	900 MHz
Distance between cluster head and receiver (D)	1,000 m

**Table 2.** BER at various SNR for different number of receive antennas

**No. of Antenna Elements**	**SNR (dB)**	**Bit Error Rate**		
		**EGC**	**MRC**	**SMC**
2	1 5 10	0.0018 0.0002 0.0000	0.0014 0.0001 0.0000	0.0016 0.0001 0.0000
3	1 5 10	1.0e–003 * [0.5900 0.0200 0.0000]	0.0006 0.0001 0.0000	1.0e–003 * [0.2240 0.0080 0.0000]
4	1 5 10	1.0e–003 * [0.2300 0.0100 0.0000]	1.0e–003 * [0.1320 0.0100 0.0000]	1.0e–003 * [0.1520 0.0120 0.0000]

**Table 3.** BER at various SNR with different number of receive antennas.

***N***_***r***_	***P***_***T***_**(W)**	**τ(μs)**	***H***_***R***_**(m)**	**SNR/BER**		
				**0**	**3**	**6**
2	10	8	2	0.000834	0.000218	2.07e–05
			5	0.000702	0.000143	9.7e–06
			10	0.000607	0.000103	6.3e–06
	10	5	10	0.000570	9.27e–05	4.8e–06
		8		0.000599	0.000103	7.6e–06
		12		0.000603	0.000104	7.7e–06
3	10	8	2	0.000409	6.97e–05	2.2e–06
			5	0.000295	3.93e–05	6e–07
			10	0.000233	2.39e–05	4e–07
	10	5	10	0.000204	1.88e–05	2e–07
		8		0.000238	0.000024	3e–07
		12		0.000249	2.43e–05	4e–07
4	10	8	2	0.000212	2.42e–05	7e–07
			5	0.000144	9.7e–06	1e–07
			10	0.000102	5.4e–06	1e–08
	10	5	10	8.95e–05	4.7e–06	1e–07
		8		0.000108	5.4e–06	3e–07
		12		0.000115	6.8e–06	4e–07
